# Phosphogypsum with Rice Cultivation Driven Saline-Alkali Soil Remediation Alters the Microbial Community Structure

**DOI:** 10.3390/plants13192818

**Published:** 2024-10-08

**Authors:** Guanru Lu, Zhonghui Feng, Yang Xu, Fachun Guan, Yangyang Jin, Guohui Zhang, Jiafeng Hu, Tianhe Yu, Mingming Wang, Miao Liu, Haoyu Yang, Weiqiang Li, Zhengwei Liang

**Affiliations:** 1 State Key Laboratory of Black Soils Conservation and Utilization, Northeast Institute of Geography and Agroecology, Chinese Academy of Sciences, Changchun 130102, China; luguanru@iga.ac.cn (G.L.); xuyang@iga.ac.cn (Y.X.); jinyangyang@iga.ac.cn (Y.J.); zhangguohui@iga.ac.cn (G.Z.); hujiafeng@iga.ac.cn (J.H.); yutianhe@iga.ac.cn (T.Y.); wangmingming@iga.ac.cn (M.W.); liumiao@iga.ac.cn (M.L.); yanghaoyu@iga.ac.cn (H.Y.); 2 College of Life Science, Baicheng Normal University, Baicheng 137000, China; fengzhonghui@bcnu.edu.cn; 3 Jilin Academy of Agricultural Sciences, Changchun 130033, China; guanfachun@163.com; 4 University of Chinese Academy of Sciences, Beijing 100049, China; 5 Jilin Da’an Agro-Ecosystem National Observation and Research Station, Da’an 131317, China

**Keywords:** phosphogypsum, rice, soil bacteria, soda saline-alkali paddy fields, Songnen Plain

## Abstract

The improvement of saline-alkali land plays a key role in ensuring food security and promoting agricultural development. Saline soils modifies the response of the soil microbial community, but research is still limited. The effects of applying phosphogypsum with rice cultivation (PRC) on soil physicochemical properties and bacterial community in soda saline-alkali paddy fields in Songnen Plain, China were studied. The results showed that the PRC significantly improved the physicochemical properties of soil, significantly reduced the salinity, increased the utilization efficiency of carbon, nitrogen, and phosphorus, and significantly increased the activities of urease and phosphatase. The activities of urease and phosphatase were significantly correlated with the contents of total organic carbon and total carbon. A redundancy analysis showed that pH, AP, ESP, HCO_3_^−^, and Na^+^ were dominant factors in determining the bacterial community structure. The results showed that PRC could improve soil quality and enhance the ecosystem functionality of soda saline-alkali paddy fields by increasing nutrient content, stimulating soil enzyme activity, and regulating bacterial community improvement. After many years of PRC, the soda-alkali soil paddy field still develops continuously and healthily, which will provide a new idea for sustainable land use management and agricultural development.

## 1. Introduction

Soil salinization is one of the important types of land degradation, which seriously restricts land-use efficiency and agricultural productivity, and has attracted significant attention worldwide [1]. According to statistics, on a global scale, the area of saline-alkali soil is about 9.54 × 10^8^ hectares [2,3]. The formation and accumulation of soil salinization are affected by geology, geomorphology, hydrology, climate, and human factors. The Songnen Plain in northeast China, as one of the three major saline-alkali soil regions in the world, with a total area of about 50,000 hectares, is a major grain-producing area and commodity grain base in China [4]. The special geographical environment of the Songnen Plain and the existence of numerous salt lakes and wetlands lead to low groundwater levels and high mineral content in the water [5], which gradually forms obstacle factors, with sodium bicarbonate and sodium carbonate as the main components. Land degradation is common, and environmental degradation is serious [1]. These factors not only limit agricultural production, but also seriously harm engineering and ecological construction, and limit sustainable agricultural, economic, and social development. As a reserve cultivated land resource for grain production, saline-alkali soil plays an extremely key role in guaranteeing national food security and expanding cultivated land resources under the background of global food crises [6].

Considerable research efforts have been devoted to the improvement and utilization of saline-alkali land worldwide. Owing to the differences in soil texture, groundwater level, climatic conditions, and ecological environment, the effects of improvement measures differ [5,7,8]. Current improvement measures include water conservancy, chemical, physical, biological, and comprehensive measures to improve the poor quality of saline-alkali soil [9,10,11]. Critical research and practical experience indicate that considering the input cost and improvement effect, the method of chemical amendment combined with rice cultivation is effective in improving soda-alkali soil and increasing grain yield. Phosphogypsum, a by-product of phosphate production and mostly composed of calcium sulfate (CaSO_4_·2H_2_O), is a soil conditioner in which dissolved Ca^2+^ can undergo a displacement reaction with Na^+^ attached to soil colloids [12]. Ca^2+^ adheres to soil colloidal surfaces and enhances soil aggregate structures. Na^+^ forms an insoluble sodium sulfate precipitate, which is discharged in irrigation and reduces soil salt content. Acidic phosphogypsum also adjusts pH, reducing alkalinity and sodium adsorption (SAR) on soda-saline soil [13,14].

Previous studies on saline-alkali land improvement mainly focused on water and salt transport, changes in soil physical and chemical properties, crop yield increase, and heavy metal pollution remediation [15,16,17,18,19]. However, studies on the impact of improving saline-alkali soil on soil biological processes are limited. As one of the main components of the soil ecosystem, soil microorganisms have significant effects on soil properties, and their composition is typically used as an index for evaluating soil quality and fertility. Soil microorganisms are also closely linked to material and energy circulation, soil structure maintenance, and soil microecological balance. Moreover, soil bacteria exhibit significant responses to changes in soil structural composition, as well as physical and chemical properties. Their activity and diversity are also closely associated with environmental stress and resource availability [20]. Additionally, various soil microbial communities can rapidly respond to changes in soil ecosystem function owing to saline-alkali soil improvement measures [7]. High salt concentrations in the soil can lead to decreased soil permeability and increased alkalinity, and directly affect microbial community characteristics, such as biomass production and activity [21,22]. Salt and alkali stress also inhibit the metabolic activity and functional diversity of soil bacteria. Particularly, high levels of salt and alkali stress can significantly reduce the utilization of carbohydrate and amino acid carbon sources by bacteria [23], alter root–oil microbial interactions, and inhibit the growth of some key bacterial groups, such as Gemmatimonas, Sphingomonas, and Bradyrhizobium, resulting in decreases in their relative abundances [24]. Rietz and Haynes (2003) also observed that soil salinization or alkalization inhibits soil enzyme activity [25], and based on their findings, Zhou et al. (2010) concluded that soil urease, alkaline phosphatase, protease, and invertase are extremely sensitive to salt stress and show reduced abundance with increasing soil salinity [26].

Compared with the extensive research on soil structure after soda saline-alkaline soil modification, research on the response of the soil microbial community to chemical modification is still limited. This study was conducted in soda saline-alkali paddy fields with different years of phosphogypsum with rice cultivation (PRC), and soil sampling was performed after the rice harvest. The aim of this study was to elucidate the bacterial mechanism of soda saline-alkali soil improvement by (1) investigating the change in physical and chemical properties of PRC-modified saline-alkali soil, (2) analyzing the diversity and composition of soil bacterial communities, and (3) establishing the relationship between soil-specific bacterial groups and soil salt, enzyme, and nutrient levels under the action of phosphogypsum. We hypothesized that PRC would significantly change soil nutrient composition and bacterial community composition and structure at different improvement years.

## 2. Results

### 2.1. Physicochemical Properties and Enzyme Activity

As the number of years of improvement increased, soil pH and EC showed decreasing trends, and the lowest values were observed in the test areas P4 and P6 (*p* < 0.05) (Table 1). Furthermore, after improvement, the SWC of the treated soil was significantly higher than that of the control soil. The TOC, TN, TP, and NH_4_-N levels also significantly increased after improvement, and their peak values were obtained for test area P6. Moreover, the highest soil AP, which was significantly higher than that obtained for test area CK, corresponded to test area P4 (Table 1); in test area P6, soil ENa and ESP significantly decreased by 82.2% and 83.67%, respectively (*p* < 0.05) (Table 1).

Furthermore, soil HCO_3_^−^, Cl^−^, Na^+^, Mg^2+^, K^+^, and Ca^2+^ levels significantly decreased after improvement (*p* < 0.05) (Table 2), and compared to test area CK, test area P6 showed 9.17% and 77.90% decreases in SO_4_^2−^ and Na^+^, respectively. Furthermore, test area P4 showed 93.33%, 81.78%, 92.07%, 80.66%, and 92.87% decreases in HCO_3_^−^, Cl^−^, Mg^2+^, K^+^, and Ca^2+^ levels, respectively, after improvement compared to test area CK.

**Figure 1:**
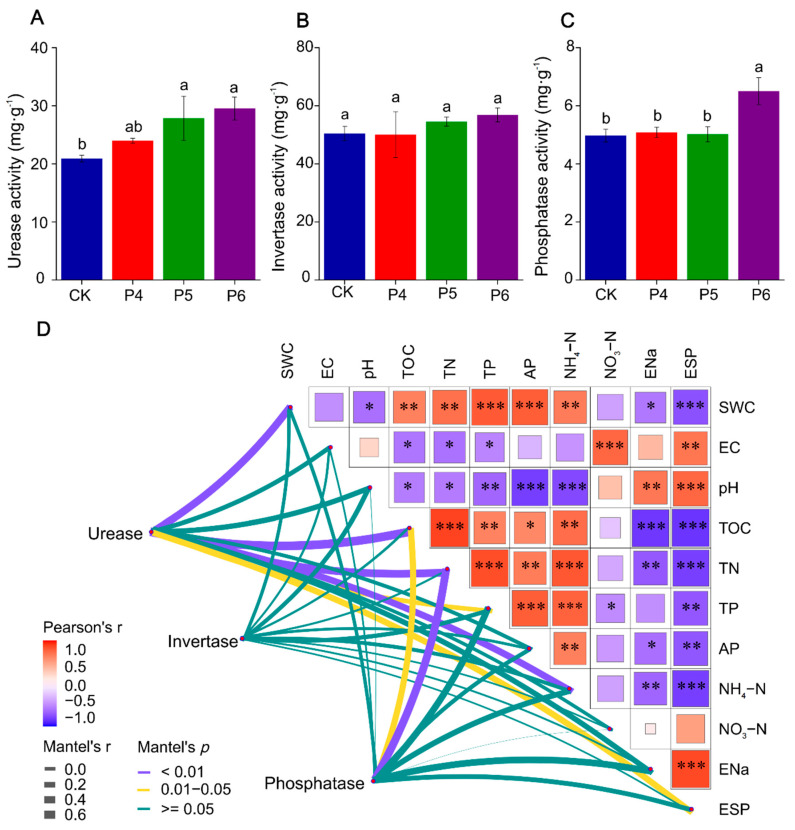
(**A**) Urease activity analyses of soil bacterial microorganisms. (**B**) Invertase activity analyses of soil bacterial microorganisms. (**C**) Phosphatase activity analyses of soil bacterial microorganisms. CK, P4, P5, and P6 indicate unmodified soda saline-alkali paddy fields and modification for 4, 5, and 6 years, respectively. Different letters indicate significant differences between different treatments (ANOVA, LSD test, *p* < 0.05). (**D**) Correlations between soil physicochemical characteristics and soil enzyme activity. The color of the correlation coefficient ranges from dark red (positive correlation) to dark blue (negative correlation). The color intensity is proportional to the correlation coefficient. The size of the grid is proportional to the absolute value of the correlation. The thickness of the connection between nodes and each environmental factor indicates the degree of correlation. The thicker the connection is, the stronger the correlation is. The color of the line between the node and the environment factor represents the significance value. SWC, soil water content; pH, potential of hydrogen; EC, electric conductivity; TOC, total organic carbon; TN, total nitrogen; TP, total phosphorus; AP, available phosphorus; NH_4_-N, nitrate; NO_3_-N, ammonium; ENa, exchangeable sodium ion; ESP, soil alkalinity. *, *p* < 0.05; **, *p* < 0.01; ***, *p* < 0.001.

As depicted in Figure 1, the activities of urease, invertase, and phosphatase in soil increased to varying degrees; the activities of urease and phosphatase under P6 treatment increased by 41.24% and 30.56%, respectively, compared with those under CK treatment, and the differences were statistically significant. Figure 1D shows the correlation analysis between soil enzyme activity and the physical and chemical properties of soil. The Mantel test results show that soil urease activity is strongly correlated with SWC, TOC, TN, and NH_4_-N (*p* < 0.05). Soil phosphatase showed a strong correlation with TOC and TN (*p* < 0.05). The correlation analysis among soil physicochemical properties showed that SWC, TOC, TN, TP, AP, and NH_4_-N were negatively correlated with EC, pH, Ena, and ESP (*p* < 0.05). However, the correlation analysis did not show any significant correlation between soil enzyme activity and soil ion content (Supporting Materials Figure S1B). The correlation analysis among ions revealed that HCO_3_^−^ and CO_3_^2−^ content were strongly positively correlated with cation content (Na^+^ Mg^2+^, K^+^, and Ca^2+^) (*p* < 0.05).

### 2.2. Bacterial Community Composition and Diversity

**Figure 2:**
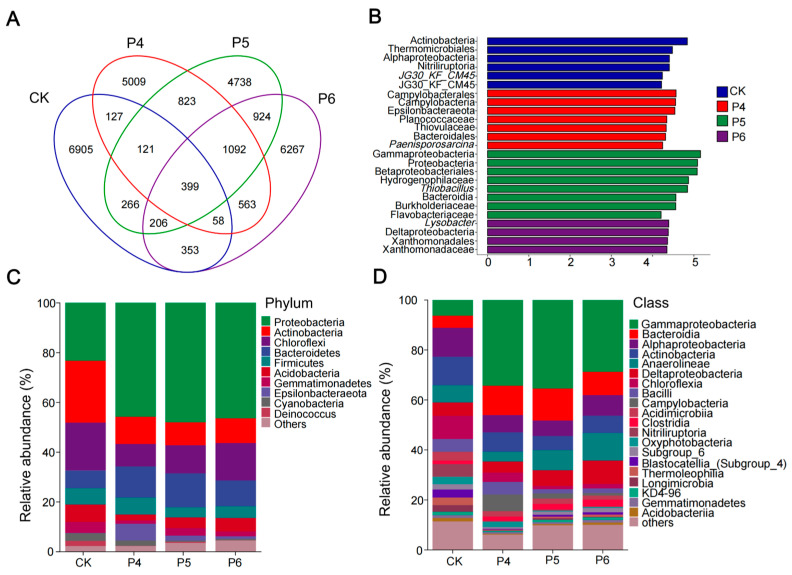
(**A**) Venn diagram representing the number of shared and unique amplicon sequence variations (ASVs) in the bacterial communities of the different experimental sites. Each ellipse represents a treatment, the overlapping area between ellipses represents shared ASVs between groups, and the number of each block represents the number of ASVs contained within the block. Microbial internal transcribed spacer amplicon sequences were clustered using the “dada2” algorithm to obtain non-monad ASVs. (**B**) Linear discriminant analysis (LDA) score. Enriched taxa with an LDA score > 4.2 are shown in the histogram. The ordinate is the classification unit with significant differences between groups, and the horizontal coordinate is a bar chart to visually display the LDA analysis logarithm scores of each classification unit. The taxa are sorted by score value size to describe their specificity within the sample grouping. Longer lengths indicate more significant differences in the taxon, and the color of the bar plot indicates the sample group corresponding to the taxon with the highest abundance. (**C**) Relative abundances of bacteria from the experimental sites at the phylum level. (**D**) Relative abundances of bacteria from the experimental sites of the first 20 most abundant class. CK, P4, P5, and P6 indicate unmodified soda saline-alkali paddy fields and modification for 4, 5, and 6 years, respectively.

There were 8435, 8192, 8569, and 9862 ASVs identified in treatments CK, P4, P5, and P6, respectively (Figure 2A). As shown in the Venn diagram, a total of 27,851 ASVs were identified in at least one of the four sites, which constituted the core fungal ASVs. There were 6905 (CK), 5009 (P4), 1154 (P5), and 6267 (P6) ASVs identified by exactly one site, which constituted the site-specific independent ASVs. The community structure at the phylum level is presented in Figure 2C. Proteobacteria (40.75%) was the dominant bacterial community in the four test sites, followed by Actinobacteria (13.74%), and Chloroflexi (13.70%), accounting for more than 60% of all sequences. The relative abundance of Proteus and Bacteroidetes was the lowest in test area CK (*p* < 0.05) and showed an increasing trend with the improvement years. Conversely, the relative abundance of Actinobacteria, Acidobacteria, and Chloroflexi was the highest in test site CK (*p* < 0.05) and showed a decreasing trend with the improvement years.

At the class level, we analyzed the top 20 bacterial genera. The most abundant in the four test sites were Gammaproteobacteria (26.11%), followed by Bacteroidia (9.66%), Alphaproteobacteria (8.25%), Actinobacteria (7.94%), Anaerolineae (7.44%), and Deltaproteobacteria (6.37%). The relative abundance of Gammaproteobacteria and Bacteroidia was the lowest in test site CK (*p* < 0.05) and increased with the improvement years. The relative abundance of Alphaproteobacteria and Anaerolineae was the highest in test site CK (*p* < 0.05), and decreased with improvement years. To identify key soil microbiota types associated with different years of modification, LEfSe analyses were performed for the CK, P4, P5, and P6 treatments (Figure 2B). Linear discriminant analysis results showed that there were 6 differential characteristics in CK (LDA > 4.2, *p* < 0.05), and the main bacterial groups were Alphaproteobacteria, Actinobacteria, Nitriliruptoria, and Thermomicrobiales. There were seven dominant bacterial groups in P4 (LDA > 4.2, *p* < 0.05), and the main bacterial groups were Epsilonbacteraeota, Bacteroidales, and Campylobacteria. There were eight dominant bacterial groups in P5 (LDA > 4.2, *p* < 0.05), and the main groups were Proteobacteria and Bacteroidia. There were four dominant bacterial groups in P6 (LDA > 4.2, *p* < 0.05), and the main groups were Deltaproteobacteria and Xanthomonadales. The main microflora was further classified and identified by evolutionary cluster analysis.

**Figure 3:**
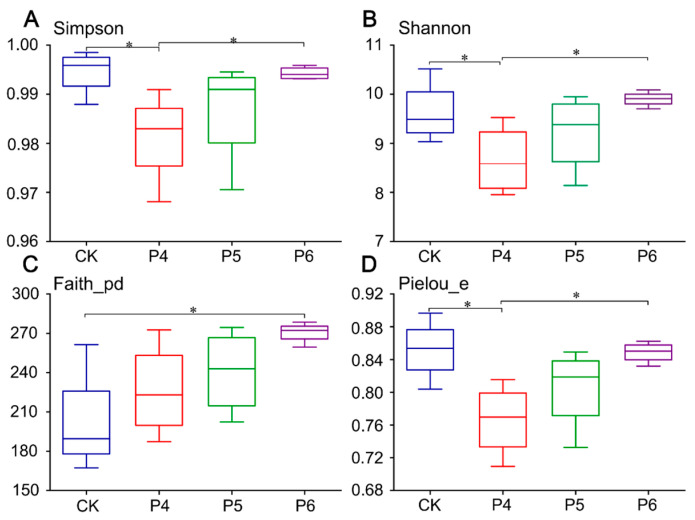
(**A**) Simpson index analyses of soil bacterial microorganisms. (**B**) Shannon index analyses of soil bacterial microorganisms. (**C**) Faith_pd index analyses of soil bacterial microorganisms. (**D**) Pielou_e index analyses of soil bacterial microorganisms. CK, P4, P5, and P6 indicate unmodified soda saline-alkali paddy fields and modification for 4, 5, and 6 years, respectively. The ends of the whiskers represent the minima and maxima, the bottom and top of the box are the first and third quartiles, respectively, and the line inside the box is the median. * indicates significant differences at the 0.05 level (ANOVA, LSD test, *p* < 0.05).

To estimate and compare soil alpha diversity and richness, we used Simpson, Shannon, Faith_pd, and Pielou_e indices (Figure 3). The Simpson’s, Shannon, and Pielou_e indices of the bacterial community were the lowest in P4 treatment, which then gradually increased. Faith_pd index was the lowest in CK treatment and showed an increasing trend with increasing years of modification.

**Figure 4:**
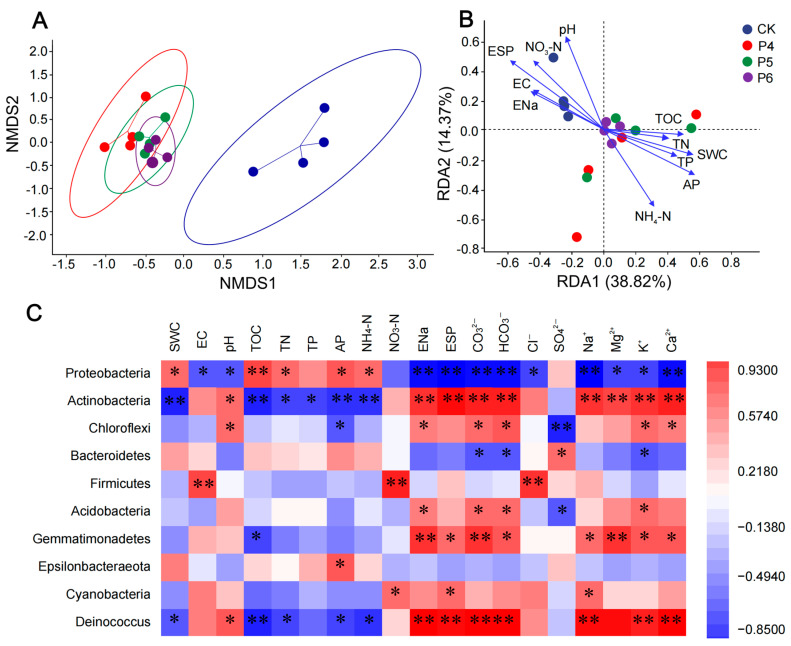
(**A**) Nonmetric multidimensional scaling plots of bacterial community structures based on Bray–Curtis distance. Each ellipse represents a treatment. Each ellipse represents a treatment. (**B**) Redundancy analysis of soil physicochemical characteristics and the microbial community structure of bacteria. This figure presents the scores of samples and significantly varied soil physicochemical characteristics on the first two axes. (**C**) Pearson correlation analysis between the relative abundance of bacterial at the phylum level and environmental factors (soil physicochemical characteristics and soil ions content). The color of the correlation coefficient ranges from dark red (positive correlation) to dark blue (negative correlation). The color intensity is proportional to the correlation coefficient. SWC, soil water content; pH, potential of hydrogen; EC, electric conductivity; TOC, total organic carbon; TN, total nitrogen; TP, total phosphorus; AP, available phosphorus; NH_4_-N, nitrate; NO_3_-N, ammonium; ENa, exchangeable sodium ion; ESP, soil alkalinity. *, *p* < 0.05; **, *p* < 0.01.

An NMDS analysis based on Bray–Curtis distance evaluated the beta diversity of bacterial communities (Figure 4A). The ANOSIM test (Supporting Materials Table S1) showed that soil with different improvement years had significantly different bacterial communities, and community distribution was significantly separated between CK and other treatments (*p* < 0.05).

### 2.3. Influences of Environmental Parameters on Bacterial Diversity and Community Structure

The results of the Pearson correlation analysis show that Shannon and Pielou_e index are significantly negatively correlated with SO_4_^2−^ (Table 3). Faith_pd index was positively correlated with SWC, TOC, TN, TP, NH_4_-N, urease, and invertase, and negatively correlated with EC, ENa, ESP, CO_3_^2−^, HCO_3_^−^, Na^+^, Mg^2+^, K^+^ and Ca^2+^.

The Pearson correlation analysis between the relative abundance of bacteria at the phylum level and environmental factors was also conducted (Figure 4C). Proteobacteria shows a strong negative correlation with EC, pH, ENa, ESP, CO_3_^2−^, HCO_3_^−^, Cl^−^, Na^+^, Mg^2+^, K^+^, and Ca^2+^, and a positive correlation with SWC, TOC, TN, AP, and NH4-N. In contrast, Actinobacteria is positively correlated with pH, ENa, ESP, CO_3_^2−^, HCO_3_^−^, Na^+^, Mg^2+^, K^+^, and Ca^2+^, and negatively correlated with TOC, TN, TP, AP, and NH_4_-N. Chloroflexi is positively correlated with pH, ENa, CO_3_^2−^, HCO_3_^−^, K^+^, and Ca^2+^, and negatively correlated with AP and SO_4_^2−^. Bacteroidetes were positively correlated with SO_4_^2−^ and negatively correlated with CO_3_^2−^, HCO_3_^−^, and K^+^. At the class level, Gammaproteobacteria showed a positive correlation with SWC, TOC, AP, and NH4-N, and a negative correlation with pH, ENa, ESP, CO_3_^2−^, HCO_3_^−^, Na^+^, Mg^2+^, K^+^, and Ca^2+^ (Supporting Materials Figure S1C). Bacteroidia showed a positive correlation with SWC and AP, but a negative correlation with pH, ENa, ESP, CO_3_^2−^, HCO_3_^−^, K^+^, and Ca^2+^. Alphaproteobacteria showed a negative correlation with SWC and AP, and a positive correlation with ESP. Actinobacteria showed a positive correlation with ENa, CO_3_^2−^, HCO_3_^−^, K^+^, and Ca^2+^.

In this study, we analyzed bacterial community structure and soil physicochemical characteristics via a redundancy analysis (RDA) (Figure 4B). Thus, we observed that the eigenvalues for the first and second axes were 38.82% and 14.37%, respectively, and the cumulative percentage variance of species data showed that the first two RDA axes explain 53.19% of the observed structural variation. Furthermore, via RDA, we identified pH, AP, and ESP (*p* < 0.05) as significant contributors to changes in bacterial community structure in all the analyzed samples (Supporting Materials Table S2). We also conducted an RDA to clarify the relationship between bacterial community structure and soil ion content. Thus, we observed that the first two axes could explain 38.22% of the observed structural variation (Supporting Materials Figure S1A). The RDA also revealed that HCO_3_^−^ and Na^+^ levels (*p* < 0.05) significantly affected the bacterial community structure in all examined samples (Supporting Materials Table S3).

## 3. Discussion

In this study, under the action of phosphogypsum, soil salinity decreased significantly. This is because, in the rice cultivation process, irrigation and drainage provide favorable conditions for the creation of dissolved Ca^2+^, promoting its movement in the vertical direction, to be replaced by the substitution Na^+^ on the soil colloid, reducing the soil viscosity and salinity, and increasing permeability. In addition, the aboveground portion and roots of rice contribute to the reduction in total alkalinity and salt in the soil and improve the quality of saline-alkali soil. In fact, the soluble salt ions in the soil are reduced to varying degrees. The reduction in the saline-alkali barrier provides a good environment for the improvement of soil fertility and the accumulation of nutrients, and the nitrogen utilization rate (NH_4_-N) and phosphorus availability (AP) of soil are significantly improved, which is also reflected in the increase in soil enzyme activity. There is additional evidence that soil enzyme activity related to nutrient acquisition is quite sensitive to increases in nitrogen concentration [27,28]. Our study also reached a similar conclusion that soil urease activity maintained a consistent trend with the level of N. Phosphatase is also an important enzyme that is involved in the hydrolysis of organophosphorus into different forms of inorganic phosphorus and is the most key biological pathway for obtaining phosphate ions from organic molecules [29]. Several previous studies have demonstrated that an increase in N can stimulate phosphatase activity [30,31]. Our findings are consistent, showing that in nitrogen-limited ecosystems, increased nitrogen stimulates microbial activity and increases demand for phosphorus, leading to an increase in phosphorus-obtaining enzymes [32].

Soil microbial diversity provides a key link for resisting environmental change and maintaining ecosystem stability, particularly in agroecosystems [33]. In this study, PRC significantly increased the diversity and phylogeny of soil bacterial communities. The reduction in soluble ions and salinity reduced the environmental stress for the survival of soil bacteria, and the accumulation of nutrients provided the available substrate for the reproduction and growth of soil bacteria. Numerous studies have shown that pH is the primary factor affecting bacterial communities in different habitats [34,35,36,37,38]. High pH conditions impose more stringent constraints on survival and fitness, exert strong selection pressures through ecological and evolutionary time, and lead to the assembly of phylogenetically more clustered bacterial communities through deterministic processes [39,40]. Soil ESP imposes physiological limitations on bacterial communities [41]. Excessively high ESP can cause great stress on bacterial communities and reduce the net growth of non-viable taxa [42]. AP plays an important role in regulating the interaction frequency of alkaline phosphomonesterase and AP transformation in the core bacterial network [43]. Evidently, the increase in biomass of the underground part of rice by the PRC also provides an energy source and constituent element for microorganisms. The improvement of the PRC undoubtedly creates a more hospitable environment for bacteria, promotes internal diversity, and enhances the ability to adapt to the external environment.

In terms of bacterial community composition, we observed that Proteus is the dominant phylum of soil bacteria under different treatments in the test area, with strong adaptability and wide distribution [44,45,46,47]. Following the increase in improvement years, the advantages of Gammaproteobacteria and Bacteroidetes gradually increased, while the relative abundance of Actinobacteria, Acidobacteria, and Chloroflexi decreased significantly. Studies have shown that increased nutrient concentrations promote more active microbial metabolism [48,49,50,51]. The increase in exogenous C redistributes resources within bacteria, and the change in the C/N ratio affects the competition between fungi and bacteria. Gammaproteobacteria and Bacteroidia are the dominant communities driving the decomposition of lignocellulose. They produce a high level of excellular enzymes that are responsive to lignocellulose, and capable of rapid colonization and recombination [52]. The improvement of PRC increased the aboveground and underground vegetation residues. The stronger decomposition ability of Gammaproteobacteria and Bacteroidia to crop stubble improved the utilization of the annual increase in carbon. However, the interaction relationships between microorganisms shift according to external conditions, and an increase in interaction always coincides with an increase in competition for resources [53]. Actinobacteria have the ability to decompose plant residues in low-fertility soils, providing a carbon source for other bacteria. However, the direct increase in nutrients reduces the advantage of Actinobacteria. Acidobacteria and Chloroflexi are oligotrophic bacteria. The low growth rate of Acidobacteria and Chloroflexi makes them lose their dominant position in environmental changes resulting from increased nutrients [54], which confirms the effect of improvement of phosphypso on improving soil fertility.

## 4. Materials and Methods

### 4.1. Study Sites and Environmental Conditions

The study site was located in the Da’an Sodic Land Ecological Experimental Station in Jilin Province, China (45°58′ N, 123°88′ E, altitude 128.5 m). The Da’an Sodic Land Ecological Experimental Station is affiliated with the Northeast Institute of Geography and Agroecology of the Chinese Academy of Sciences. This area experiences a temperate semi-humid and semi-arid monsoon climate, which is a typical temperate continental climate situated at an altitude of 150 and 200 m. The average annual temperature is 4.7 °C. The annual evaporation is between 1250 and 1650 mm, and annual precipitation is between 370 and 400 mm.

### 4.2. Experimental Treatments and Sampling

In November 2021, four treatments of phosphogypsum with rice cultivation unimproved (CK), four years of improvement (P4), five years of improvement (P5), and six years of improvement (P6) in the planting area were selected for 0–15 cm soil layer sampling. Field management was consistent across all treatments. Four plots were selected for each treatment, with rows separating the plots. Each plot was sampled using the five-point method, mixed evenly, and divided into three parts. One part was air-dried to determine the physical and chemical properties of the soil, another part was placed at 4 °C to detect enzyme activity, and the third remaining part was placed in dry ice and transferred to −80 °C for microbial sequencing.

### 4.3. Soil Physicochemical Properties and Enzyme Analysis

Soil water content (SWC) was measured using the drying method. EC and pH values were measured using the supernatant by employing a 1:5 (soil:water) solution (*w*/*v*) with a conductivity meter (DDS-307A, Shanghai Leici, Shanghai, China) and a pH meter (PHS-3E, Shanghai Leici, Shanghai, China). Total organic carbon (TOC) was evaluated with a TOC analyzer (TOC-LCPH, Shimadzu, Kyoto, Japan) using a 1:4 (soil:water) solution (*w*/*v*). Nitrate (NO_3_-N) and ammonium (NH_4_-N) were extracted with 2 M potassium chloride, and available phosphorus (AP) was extracted with 0.5 M sodium bicarbonate. The measurements were measured using a continuous flow analyzer (SKALAR, Bread, The Netherlands). Total phosphorus (TP) was also determined using the continuous flow analyzer after digestion of the soil sample. Total nitrogen (TN) was determined using an elemental analyzer (Elemental Vario MACRO, Frankfurt, Germany). The exchangeable sodium ions (ENa) in the soil were measured using the ammonium acetate-ammonium hydroxide exchange method, while the soil cation exchange capacity (CEC) was determined using the ammonium chloride-acetic acid exchange method. Soil alkalinity (ESP) was calculated using the formula:

ESP = (ENa/CEC) × 100%

The content of Ca^2+^, Mg^2+^, Na^+^, and K^+^ in the soil were detected using an inductively coupled plasma emission spectrometer (TOC-LCPH, Shimadzu, Kyoto, Japan). CO_3_^2−^ and HCO_3_^−^ ions were neutralized using a double indicator method (phenolphthalein and methyl orange). Cl^−^ ions were measured using silver nitrate titration, while SO_4_^2−^ ions were measured using barium chloride titration. Urease activity was measured 24 h after culturing the soil with urea at 37 °C and measured spectrophotometrically at 578 nm. Phosphatase activity was expressed as the mass of phenol released per gram of dry soil and was measured spectrophotometrically at 570 nm. Invertase activity was expressed as the mass of glucose released per gram of dry soil and was measured spectrophotometrically at 508 nm [55].

### 4.4. Microbial Diversity Analysis

Total genomic DNA samples were extracted using the OMEGA Soil DNA Kit (M5635-02) (OMEGA Bio-Tek, Norcross, GA, USA) and stored at −20 °C. The quantity and quality of extracted DNA were evaluated using a NanoDrop NC2000 spectrophotometer (Thermo Fisher Scientific, Waltham, MA, USA) and agarose gel electrophoresis, respectively. The highly variable V3-V4 region of the bacterial 16S rRNA gene with a length of about 468bp was selected for sequencing. The 16S rRNA gene was amplified using specific primers V3 and V4 (F: ACTCCTACGGGAGGCAGCA R: TCGGACTACHVGGGTWTCTAAT). PCR amplicons were purified using Vazyme VAHTSTM DNA Clean Beads (Vazyme, Nanjing, China). Quantification was performed using the Quant-iT PicoGreen dsDNA Assay Kit (Invitrogen, Carlsbad, CA, USA). The results were amplified by 2% agarose gel electrophoresis; the target fragments were cut and then recovered with the Axygen gel recovery kit (Corning Inc., Corning, NY, USA). After individual quantification, amplicons were pooled in equal amounts and pair-end 2 × 250 bp sequenced on an Illumina MiSeq platform using the MiSeq Reagent Kit v3 (Illumina, San Diego, CA, USA) at Shanghai Personal Biotechnology Co., Ltd. (Shanghai, China).

Microbiome bioinformatics used QIIME2 2019.4 [56] according to the official tutorial (https://docs.qiime2.org/2019.4/tutorials/, accessed on 13 September 2023) for fine-tuning. The non-single fragment amplicon sequence variation (ASV) was obtained using the dada2 algorithm to cluster the microorganism transcriptional spacer amplicon sequences. Matt was used for amplicon sequence variation alignment [57], and fasttre2 was used to construct phylogeny [58]. The diversity plug-in was used to estimate the alpha-diversity (Simpson, Shannon, Faith_pd, and Pielou_e) and beta-diversity (Bray–Curtis dissimilarity) indicators, and the sample was rare to a consistent number of sequences per sample.

### 4.5. Statistical Analysis

Alpha diversity metrics were used to analyze species diversity, including the Simpson’s, Shannon’s, Faith_pd’s, and Pielou_e’s indices. These indices were calculated using QIIME2 (Version 2019.4) and visualized using R software (Version 2.15.3) (http://www.r-project.org). To assess fungal community structure, beta diversity was analyzed using the non-metric multidimensional scaling method based on Bray–Curtis distance metrics and executed via the vegan package in R software. An analysis of similarities (ANOSIM) was performed using the anosim function in the vegan package, and significant differences between groups were assessed using Bray–Curtis distance values.

Fisher’s LSD method was used to test differences between treatments. A correlation analysis was conducted between the main parameters of soil properties and microbial communities. The vegan package [59] in the R 3.5.2 software (R Core Team, 2019) was used to perform a redundancy analysis (RDA) on the effects of environmental factors on soil microbial communities, while the ggplot2 software package was used to draw RDA graphics. All data are presented as the mean and standard error from four repeated measurements, and significance was assessed at the 5% level. Fisher’s LSD and correlation analyses were performed using SPSS 24.0 (SPSS Inc., Chicago, IL, USA). A linear discriminant analysis Effect size (LEfSe) analysis was performed using the “ggtree” function in the “vegan” package of the R software (R Foundation for Statistical Computing, Vienna, Austria). After the correlation analysis and mantel test of soil enzyme activity and physicochemical properties, the “linkET” package of R was used to display the correlation between omics, and the correlation between omics in the form of a correlation heatmap and network map.

## 5. Conclusions

**Figure 5:**
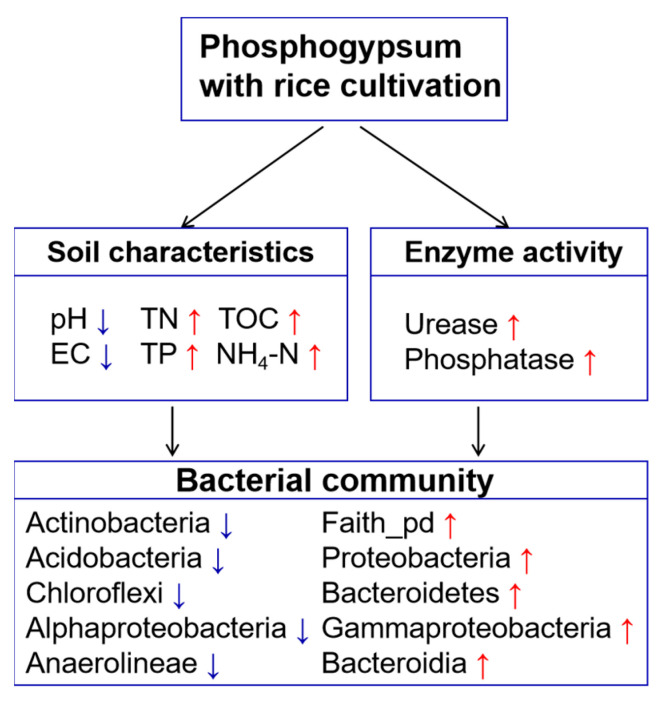
This model illustrates the mechanism of the improvement of phosphogypsum with rice cultivation on soil quality and bacterial community in paddy field. PRC significantly reduced soil salinity (pH, EC) and increased soil fertility (TOC, TN, TP, NH_4_-N, Urease, Phosphatase). In addition, bacterial diversity and community composition also changed significantly, Faith_pd index increased significantly, and the relative abundance of Proteobacteria, Bacteroidetes, Gammaproteobacteria, and Bacteroidia increased. The relative abundance of Actinobacteria, Acidobacteria, Chloroflexi, Alphaproteobacteria, and Anaerolineae decreased. The reduction in salt pressure and the increase in nutrients provide more suitable conditions for bacterial reproduction and habitat and promote the activity of beneficial bacteria. This helps to improve the ability of bacterial decomposition, strengthen material circulation, and improve soil quality. The red arrow up and the blue arrow down represent the increase and decrease in the corresponding parameter. TOC, total organic carbon; TN, total nitrogen; TP, total phosphorus; NH_4_-N, nitrate.

Phosphogypsum with rice cultivation has obvious advantages for the improvement of soda-saline soil. Specifically, the application of phosphogypsum with rice cultivation can effectively alleviate the problem of soil salinization and significantly improve the nutrient content of the soil (Figure 5). These factors work together to improve soil fertility and lead to positive changes in the diversity and structure of bacterial communities. Further analyses showed that pH, AP, ESP, HCO_3_^−^ and Na^+^ were the main environmental factors that shaped the structure of the bacterial community. Given the central role of soil microbial communities in driving multiple ecosystem functions and ecological processes, changes in the structure of soil bacterial communities will be critical to maintaining productivity. These results help to fill in the gaps in the microbial mechanisms for the sustainable restoration of saline-alkali land and provide scientific support for the study of microbial community changes on time series. These findings help to fill in the gaps in the microbial mechanisms for sustainable remediation of soda-alkaline land. The findings also provide strong scientific support for the sustainable use of soda saline-alkali soil resources and for promoting agricultural development.

## Figures and Tables

**Table 1:** Soil physicochemical characteristics at the different experimental sites of different treatments.

	CK	P4	P5	P6
SWC (%)	15.44 ± 0.44 b	20.43 ± 1.40 a	19.96 ± 1.35 a	20.11 ± 1.65 a
pH	10.14 ± 0.10 a	8.47 ± 0.10 b	9.01 ± 0.37 b	9.02 ± 0.21 b
EC (μS·cm^−1^)	1092.75 ± 437.97 a	642.75 ± 75.25 b	667.75 ± 248.15 b	343.00 ± 16.25 c
TOC (%)	0.31 ± 0.03 b	0.50 ± 0.04 a	0.51 ± 0.10 a	0.63 ± 0.04 a
TN (mg·kg^−1^)	315.05 ± 30.12 b	513.35 ± 37.23 ab	543.76 ± 95.45 ab	692.39 ± 104.14 a
TP (mg·kg^−1^)	334.53 ± 30.74 b	460.52 ± 34.52 a	448.40 ± 21.30 a	474.92 ± 50.89 a
AP (mg·kg^−1^)	8.01 ± 0.99 b	37.13 ± 5.99 a	23.78 ± 3.22 a	25.07 ± 5.35 a
NH_4_-N (mg·kg^−1^)	2.20 ± 0.21 b	3.74 ± 0.26 a	3.84 ± 0.42 a	4.03 ± 0.42 a
NO_3_-N (mg·kg^−1^)	13.75 ± 8.25 a	3.82 ± 2.31 a	3.07 ± 1.21 a	9.15 ± 2.49 a
ENa (cmol·kg^−1^)	5.73 ± 1.33 a	1.98 ± 0.33 b	2.60 ± 0.82 b	1.02 ± 0.22 b
ESP (%)	39.37 ± 4.11 a	11.09 ± 1.65 b	13.88 ± 4.38 b	6.43 ± 1.83 b

SWC, soil water content; EC, electric conductivity; TOC, total organic carbon; TN, total nitrogen; TP, total phosphorus; AP, available phosphorus; NH_4_-N, nitrate; NO_3_-N, ammonium; ENa, exchangeable sodium ion; ESP, soil alkalinity. CK, P4, P5, and P6 indicate unmodified soda saline-alkali paddy fields and modification for 4, 5, and 6 years, respectively. Different letters indicate significant differences between different treatments (ANOVA, LSD test, *p* < 0.05).

**Table 2:** Soil ion content at the different experimental sites of different treatments.

	CK	P4	P5	P6
CO_3_^2−^ (mg·kg^−1^)	401.40 ± 137.76	nd	nd	nd
HCO_3_ (mg·kg^−1^)	8368.96 ± 1580.67 a	558.15 ± 181.39 b	1720.20 ± 711.95 b	861.93 ± 188.30 b
Cl^−^ (mg·kg^−1^)	628.35 ± 320.32 a	114.49 ± 3.66 b	119.81 ± 9.54 b	129.58 ± 12.17 b
SO_4_^2−^ (mg·kg^−1^)	423.64 ± 291.41 b	1982.52 ± 472.99 a	1302.62 ± 547.86 ab	384.78 ± 46.78 b
Na^+^ (mg·kg^−1^)	1962.41 ± 379.77 a	899.66 ± 79.29 b	869.54 ± 274.02 b	433.79 ± 41.65 b
Mg^2+^ (mg·kg^−1^)	330.99 ± 125.55 a	49.59 ± 27.71 b	71.01 ± 27.59 b	26.23 ± 9.10 b
K^+^ (mg·kg^−1^)	44.34 ± 12.11 a	10.64 ± 1.68 b	13.44 ± 3.94 b	8.58 ± 0.98 b
Ca^2+^ (mg·kg^−1^)	1885.96 ± 361.70 a	219.17 ± 73.79 b	405.43 ± 156.89 b	134.43 ± 33.17 b

CK, P4, P5, and P6 indicate unmodified soda saline-alkali paddy fields and modification for 4, 5, and 6 years, respectively. Different letters indicate significant differences between different treatments (ANOVA, LSD test, *p* < 0.05); nd stands for not detected.

**Table 3:** Pearson correlation analysis of bacterial alph-diversity and environmental factors (soil physicochemical characteristics, soil ions content and soil enzyme activity) at the experimental sites of different treatments.

	Simpson	Shannon	Faith_pd	Pielou_e
SWC (%)	−0.131	0.030	0.596 *	−0.147
pH	0.146	0.137	−0.424	0.263
EC (μS·cm^−1^)	−0.102	−0.326	−0.579 *	−0.211
TOC (%)	0.012	0.264	0.732 **	0.066
TN (mg·kg^−1^)	0.109	0.322	0.685 *	0.160
TP (mg·kg^−1^)	−0.038	0.123	0.552 *	−0.029
AP (mg·kg^−1^)	−0.371	−0.249	0.362	−0.405
NH_4_-N (mg·kg^−1^)	−0.042	0.038	0.500 *	−0.089
NO_3_-N (mg·kg^−1^)	0.118	0.052	−0.155	0.074
ENa (cmol·kg^−1^)	0.167	−0.070	−0.701 **	0.146
ESP (%)	0.164	−0.044	−0.696 **	0.150
CO_3_^2−^ (mg·kg^−1^)	0.322	0.106	−0.617 *	0.328
HCO_3_^−^ (mg·kg^−1^)	0.265	0.097	−0.632 **	0.304
Cl^−^ (mg·kg^−1^)	0.036	−0.073	−0.435	0.010
SO_4_^2−^ (mg·kg^−1^)	−0.395	−0.605 *	−0.274	−0.583 *
Na^+^ (mg·kg^−1^)	0.047	−0.237	−0.794 **	−0.036
Mg^2+^ (mg·kg^−1^)	0.222	0.010	−0.636 **	0.233
K^+^ (mg·kg^−1^)	0.271	0.139	−0.515 *	0.335
Ca^2+^ (mg·kg^−1^)	0.235	0.044	−0.677 **	0.263
Urease (mg·g^−1^)	0.281	0.385	0.669 **	0.257
Invertase (mg·g^−1^)	−0.067	0.159	0.382	−0.015
Phosphatase (mg·g^−1^)	0.176	0.389	0.572 *	0.261

SWC, soil water content; EC, electric conductivity; TOC, total organic carbon; TN, total nitrogen; TP, total phosphorus; AP, available phosphorus; NH_4_-N, nitrate; NO_3_-N, ammonium; ENA, exchangeable sodium ion; ESP, soil alkalinity. CK, P4, P5, and P6 indicate unmodified soda saline-alkali paddy fields and modification for 4, 5, and 6 years, respectively. *, *p* < 0.05; **, *p* < 0.01.

## Data Availability

The original contributions presented in the study are included in the article/Supplementary Materials, further inquiries can be directed to the corresponding authors.

## Supplementary Materials

The following are available online at https://www.mdpi.com/article/10.3390/plants13192818/s1, Table S1. Anosim test performed on the experimental sites using bacteria community structure; Table S2. Redundancy analysis and permutation test performed on the experimental sites using bacteria community structure and soil physicochemical characteristics; Table S3. Redundancy analysis and permutation test performed on the experimental sites using bacteria community structure and soil ion content; Figure S1. (A) Redundancy analysis of soil ion content and the microbial community structure of bacteria. (B) Correlations between soil ion content and soil enzyme activity. (C) Pearson correlation analysis between the relative abundance of the first 20 most abundant class and environmental factors (soil physicochemical characteristics and soil ions content).
