# Supplementary material for: Phosphogypsum with Rice Cultivation Driven Saline-Alkali Soil Remediation Alters the Microbial Community Structure
Source: Plants (Basel). 2024 Oct 8;13(19):2818. doi: 10.3390/plants13192818 (PMC11479165; doi:10.3390/plants13192818)
Supplement: Supplementary file 1 [file plants-13-02818-s001.zip › plants-3234396-supplementary.pdf]

Supplementary material

Table S1. Anosim test performed on the experimental sites using bacteria community structure.

Group	R	p-value	q-value
all	0.460938	0.002	-
CK-P4	1	0.03	0.054
CK-P5	0.947917	0.029	0.054
CK-P6	0.90625	0.025	0.054
P4-P5	0.03125	0.574	0.574
P4-P6	0.395833	0.036	0.054
P5-P6	0.114583	0.145	0.174

CK, P4, P5 and P6 indicate unmodified soda saline-alkali paddy fields and modification for 4, 5, and 6 years, respectively.

Table S2. Redundancy analysis and permutation test performed on the experimental sites using bacteria community structure and soil physicochemical characteristics.

	RDA1	RDA2	r^2	Pr(>r)
SWC	0.958658494	-0.284559118	0.318079897	0.077
EC	-0.864612832	0.502438703	0.24654039	0.194
pH	-0.353302306	0.935509209	0.422872932	0.028
TOC	0.997671972	-0.068195579	0.230404861	0.169
TN	0.990675297	-0.136244104	0.151702862	0.317
TP	0.93525701	-0.353969386	0.218052299	0.193
AP	0.885377081	-0.464873557	0.391278952	0.04
NH_4-N	0.522666194	-0.852537418	0.323993602	0.066
NO_3-N	-0.693813087	0.720155122	0.389503681	0.081
ENa	-0.878319745	0.478073661	0.257825417	0.142
ESP	-0.789430663	0.613839741	0.529519514	0.007

SWC, soil water content; EC, electric conductivity; TOC, total organic carbon; TN, total nitrogen; TP, total phosphorus; AP, available phosphorus; NH_4-N, nitrate; NO_3-N, ammonium; ENa, exchangeable sodium ion; ESP, soil alkalinity.

Table S3. Redundancy analysis and permutation test performed on the experimental sites using bacteria community structure and soil ion content.

	RDA1	RDA2	r^2	Pr(>r)
CO_3^{2-}	-0.946890988	0.321554749	0.418079321	0.076
HCO_3^-	-0.909903205	0.414820632	0.437991362	0.041
Cl^-	-0.929359777	0.369175303	0.391352638	0.067
SO_4^{2-}	0.408527716	-0.912745915	0.303488169	0.104
Na^+	-0.951058988	0.309009386	0.401916774	0.036
Mg^{2+}	-0.996675133	0.081478086	0.246267083	0.152
K^+	-0.973553855	0.228457635	0.236836118	0.161
Ca^{2+}	-0.966388803	0.257084968	0.397279867	0.067

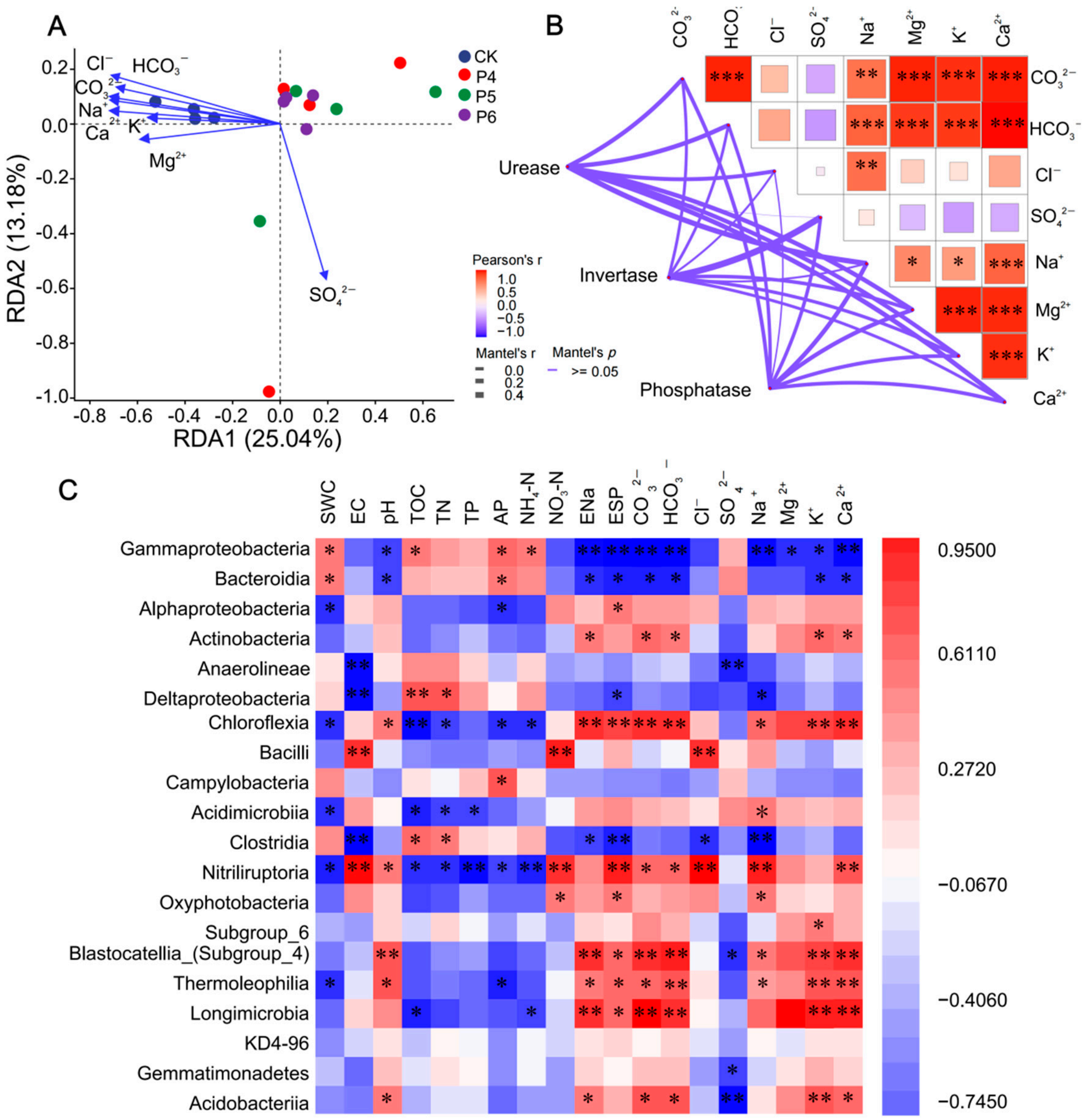

Figure S1. (A)Redundancy analysis of soil ion content and the microbial community structure of bacteria. The figure presents the scores of samples and significantly varied soil ion content on the first two axes. (B)Correlations between soil ion content and soil enzyme activity. The color of the correlation coefficient ranges from dark red (positive correlation) to dark blue (negative correlation). The color intensity is proportional to the correlation coefficient. The size of the grid is proportional to the absolute value of the correlation. The thickness of the connection between nodes and each environmental factor indicates the degree of correlation. The thicker the connection is, the stronger the correlation is. The color of the line between the node and the environment factor represents the significance value. (C)Pearson correlation analysis between the relative abundance of the first 20 most abundant class and environmental factors (soil physicochemical characteristics and soil ions content). The color of the correlation coefficient ranges from dark red (positive correlation) to dark blue (negative correlation). The color intensity is proportional to the correlation coefficient. SWC, soil water content; pH, potential of hydrogen; EC, electric conductivity; TOC, total organic

carbon; TN, total nitrogen; TP, total phosphorus; AP, available phosphorus; NH_4-N, nitrate; NO_3-N, ammonium; ENa, exchangeable sodium ion; ESP, soil alkalinity. *, $P < 0.05$; **, $P < 0.01$.
